# A CMIP6 multi-model based analysis of potential climate change effects on watershed runoff using SWAT model: A case study of kunhar river basin, Pakistan

**DOI:** 10.1016/j.heliyon.2024.e28951

**Published:** 2024-04-10

**Authors:** Abdul Waheed, Muhammad Hidayat Jamal, Muhammad Faisal Javed, Khairul Idlan Muhammad

**Affiliations:** a Department of Civil Engineerig, COMSATS Unversity Abbottabad, KPK, Pakistan; b Department of Civil Engineering, UTM, Malaysia; c Department of Civil Engineering, Ghulam Ishaq Khan Institute of Engineering Sciences and Technology, Topi, Swabi, Khyber Pakhtunkhwa, 23640, Pakistan

**Keywords:** Climate change, SWAT model, Kunhar river basin, Climate projections, Hydropower development, Trend analysis

## Abstract

The hydrological regimes of watersheds might be drastically altered by climate change, a majority of Pakistan's watersheds are experiencing problems with water quality and quantity as a result precipitation changes and temperature, necessitating evaluation and alterations to management strategies. In this study, the regional water security in northern Pakistan is examined about anthropogenic climate change on runoff in the Kunhar River Basin (KRB), a typical river in northern Pakistan using Soil and Water Assessment tool (SWAT) and flow durarion curve (FDC). Nine general circulation models (GCMs) were successfully utilized following bias correction under two latest IPCC shared socioeconomic pathways (SSPs) emission scenarios. Correlation coefficients (R^2^), Nash-Sutcliffe efficiency coefficients (NSE), and the Percent Bias (PBIAS) are all above 0.75. The conclusions demonstrate that the SWAT model precisely simulates the runoff process in the KRB on monthly and daily timescales. For the two emission scenarios of SSP2-4.5 and SSP5-8.5, the mean annual precipitation is predicted to rise by 3.08 % and 5.86 %, respectively, compared to the 1980–2015 baseline. The forecasted rise in mean daily high temperatures is expected to range from 2.08 °C to 3.07 °C, while the anticipated increase in mean daily low temperatures is projected to fall within the range of 2.09 °C–3.39 °C, spanning the years 2020–2099. Under the two SSPs scenarios, annual runoff is estimated to increase by 5.47 % and 7.60 % due to climate change during the same period. Future socioeconomic growth will be supported by a sufficient water supply made possible by the rise in runoff. However, because of climate change, there is a greater possibility of flooding because of increases in both rainfall and runoff. As a result, flood control and development plans for KRB must consider the climate change's possible effects. There is a chance that the peak flow will move backwards relative to the baseline.

## 1 Introduction

Water shortage has been a concern for Pakistan for decades, and it is only going to get worse due to the climate change and inadequate water management. Climate change is making floods worse in the lower Indus basin, especially in Sindh province of Pakistan, due to the convergence of river flows with the summer and wet seasons Akhter et al. [1]. The careful monitoring of northern catchments is essential as a substantial amount of river water originates from the melting of the cryosphere. In addition, organizing and implementing hydrological structures requires constant monitoring and supervision of climate change's effects on water supplies [2]. The anticipated increase in global temperature has the potential to impact hydrological cycles, leading to potential disruptions in the existing hydrological system [3]. Global average surface temperatures, precipitation patterns, and the frequency of natural disasters like floods and droughts have experienced a significant increase. The Fourth Assessment Report (AR4) by the Intergovernmental Panel on Climate Change (IPCC) indicates that this upward trend is anticipated to continue [4]. The IPCC's sixth assessment report issues an urgent warning that in the next two decades, global temperature will increase by 1.5 °C, and only significant reductions in carbon emissions from now can prevent a global environmental disaster. Greenhouse gas (GHG) emissions caused by humans have recently hit record peak levels. The burning of substantial amounts of fossil fuels led to the occurrence of disasters such as the Siberian heat wave in 2020 and the Asian heat wave in 2016 [5]. Climate change-induced increases in surface air temperatures lead to shifts in rainfall patterns, consequently impacting the global water supply. More water is released into the sky with the increase in earth's temperature which spikes the frequency of extreme weather events. For each degree Celsius increase in temperature, the air can produce about 4 % more water, resulting in heavy rainfall [6].

German Watch placed Pakistan in the top ten nations most vulnerable to climate change. (Pakistan Economic Survey 2019–20). A violent flooding hit Pakistan and India in the month of April 2010 just after an enormous heat wave Pakistan experienced a slew of abnormal weather events in year (2022). Following winter, there were four heat waves that led to a year with no discernible spring and caused damage to crops due to scorching. Recent rainfalls in several parts of Pakistan have been recorded about four times higher than the past 30-years rainfall average.. . .

The water issues in Pakistan represent a major challenge for the country's water resource management and policymakers [7]. Pakistan's average annual water supply was 5050 m^3^ in 1952 and has reduced to 1100 m^3^ in 2006 [8]. Climate change severely affects hydropower and irrigation systems. For hydropower plants to run smoothly, runoff rivers need a consistent water supply [9]. Due to climate change, it is expected that there will be variations in the regional and temporal flow patterns within the system, which will significantly impact the cost efficiency of these projects [10].

Kunhar River Basin (KRB), the subject of the present research, is an important northern catchment of Pakistan feeding domestic and irrigation sectors, a tributary of the Mangla River, which serves as the water source for five Hydropower projects (HPPs). This study utilized the SWAT model [11] and nine downscaled, bias-corrected General Circulation Models (GCMs) to explore the impact of climate changes on discharge in the KRB. Two Shared Socioeconomic Pathway (SSP) scenarios SSP2-4.5 and SSP5-8.5, were taken into consideration during the analysis. Previous studies on Kunhar River Basin (KRB) includes Akhter et al. [1] which predicted an increase in both annual precipitation (by up to 16 %) and temperature (by up to 4.8 °C). Babur et al. (2016) [12] utilized a SWAT model to analyze the KRB. Streamflow projections were simulated for three timeframes were compared with the baseline period of 1981–2010 under both RCP 4.5 and RCP 8.5 scenarios. The results indicated an overall increase in mean annual flow, with the highest inflow observed during the winter and spring, and the lowest inflow recorded in the summer and autumn. Mahmood and Babel (2016) [13], developed HEC-HMS model and the results were assessed in comparison to the average streamflow. The average yearly flow was observed to rise equally in the A2 and B2 scenarios while winter and spring show a declining tendency, summer and fall have a clear rising trend.

There are three runoff river Dams in planning phase, one is under construction (883 MW Suki Kinari HPP), and one is in operational (147 MW Patrind HPP) on River Kunhar, which flows from the Greater Himalayas and joins the Jhelum River to form the Mangla Reservoir. River Kunhar has great potential for power production and irrigation. Sediment transport has been studied in the Kunhar river basin previously [14], and changes in runoff due to climate change have been assessed in only a few studies Ali and Shah, 2015 [15], (Mahmood et al., 2016 [13]; Haseeb Akbar, 2020 [16]). The recent climate fluctuations, evident through early heatwaves in March and April 2022, and the unprecedented and destructive floods, especially in the arid regions of Pakistan, emphasize the critical necessity for a renewed investigation into the KRB.

For peak efficiency, run-of-river hydropower systems depend on a steady and substantial water supply. The financial viability of these projects is at risk due to climate change intensifying the spatial and temporal inconsistency of the system's flow [17]. However, studies have produced inconsistent results when trying to predict whether or not rain levels will increase. Ali and Shah (2015) [15] used a snowmelt-runoff model to examine the Kunhar River basin (KRB). They discovered that temperatures have been observed to increase by 2 °C, rainfall by 20 %, and discharge volume by 27 % by the turn of the twenty-first century. Mean monthly temperatures for the Mangla watershed show a tendency that is both seasonal and intra-annual in nature. In spring, the mean temperature series goes up, during the cooler months of fall and winter, it increases, but during the warmer summer months, it drops [12]. All previous studies on KRB used not more than two GCMs to predict future runoff under changing climate. Weghost (1996) [18], Mukhopadyay (2015) [19], Lv,Z. et al. (2020) [20], Adnaan,M. et al. (2017) [21] and Musiee et al. (2020) [22] all agree that hydrologic models are necessary for efficient water resource management. Reggiani et al. (2017) [23] differentiated between energy-based and temperature-based hydrological models, each requiring a distinct set of data for conducting simulations. The accuracy of future hydrological modelling scenario predictions relies primarily on the precision of the meteorological model employed as input. Resolution-wise, RCP scenarios are superior to their SRES counterparts, and they address many of their limitations [24].

After comprehensive literature review and applicability of CMIP6 in Pakistan [[25], [26], [27]], nine CMIP6 global climate models were selected to use in this study for comparison based on their spatial resolution: cams-csm1-0, cnrm-esm2-1, fgoals-g3, ec-earth3-veg, ipsl-cm6a-lr, gfdl-esm4, miroc6,mri-esm2-0, ukesm1-0-ll; and two SSP emission scenarios were used to simulate future discharge: SSP2-4.5, and SSP5-8.5. The CMIP6 climate models used for Pakistan were found to have strong applicability in previous studies. Although the extensional forecasting capacity of GCMs for future meteorological aspects is excellent, the model's spatial resolution is limited. Accurate projections of climate change's effects on regional watersheds require general circulation models (GCMs) to address the problems of downscaling and bias correction. Scaling down the outcomes of general circulation model simulations, conducted at a global scale and low resolution, to provide local, high-resolution regional climate information is an efficient approach to reconcile the scale disparity inherent in climate models [28]. For bias correction, the Linear Scaling (LS) in CMhyd software method was used. The LS method involves developing a linear link between climate model output and observed data. Most researchers are combining results from GCMs and watershed-scale hydrological models like SWAT to find out how climate change may affect hydrological processes. Previous research has successfully applied to the strategy of integrating the SWAT with global climate models to predict how global warming will affect certain river basins. The CA-Markov model was applied to simulate potential temperature scenarios and LULC in order to predict how the Luo River Basin might react to climate change [29]. Researchers established a significant association between variations in rainfall and changes in runoff, along with a negative correlation between runoff and temperature fluctuations. Moreover, they noted that the correlation between LULC, climate change and runoff is non-linear. Clina et al. [30] assessed possible consequences on runoff management and water supply in the watershed of Lagena de Sauce. Zhao,P et al. [31] employed three GCMs under two RCP scenarios to analyze the development of droughts in the Weihe watershed using SWAT.

Considering the existing gap in the literature and the significance of the research in the context of multiple Sustainable Development Goals (SDGs), the goals of this study are to investigate the impact of climate change on the streamflow in the Kunhar River Basin (KRB), North Pakistan. Soil and Water Assessment Tool (SWAT) that has been applied for evaluation of water resource potential under climate change and CORDEX- South Asia climate model SSP outputs of mid-range (SSP2-4.5) and high-level (SSP5-8.5) were used.

## 2 Data and methodology

### 2.1 Study area and data

**Fig. 1:**
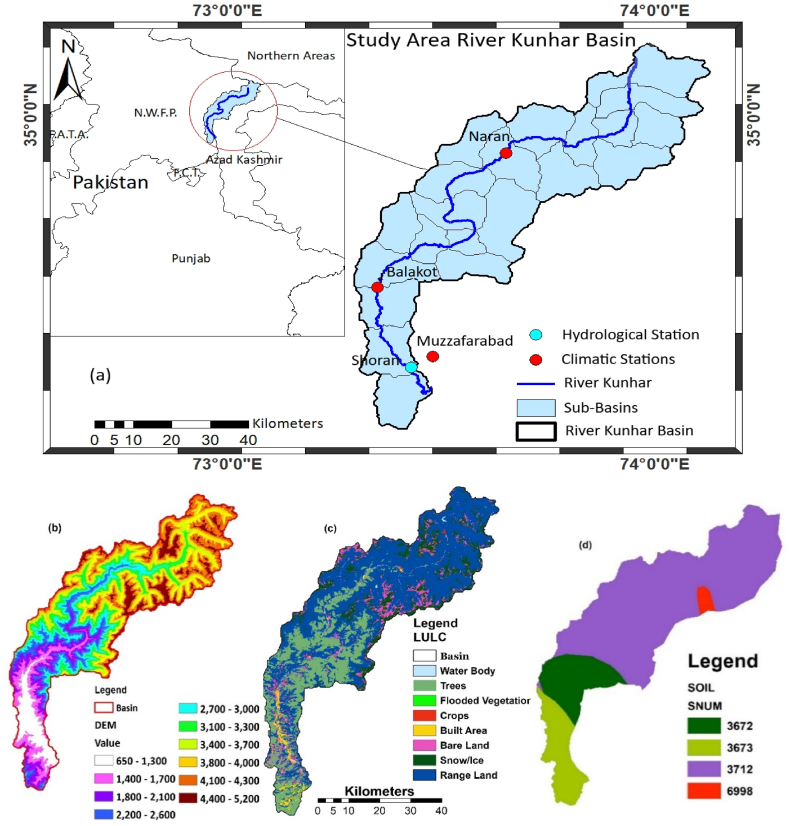
SWAT data inputs. (a) Area map, (b) DEM, (c) LULC (d) Soil classifications of the KRB.

**Fig. 2:**
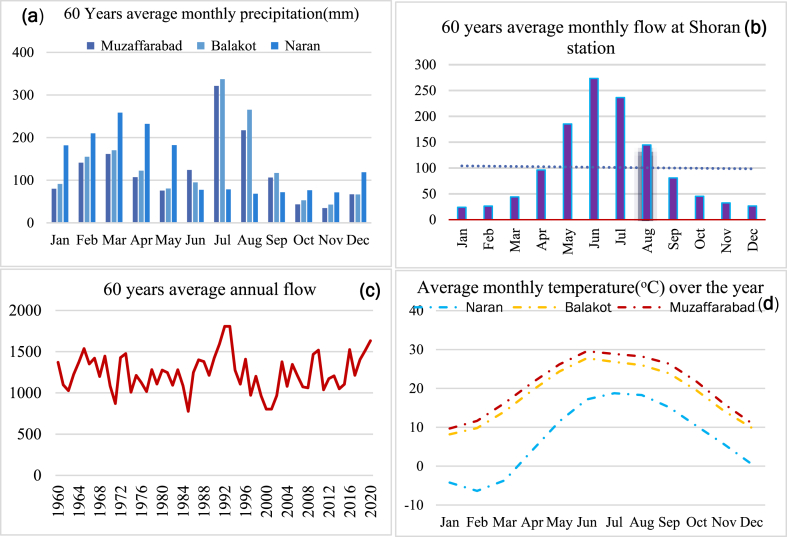
Average fluctuations over the year in KRB a) Average monthly PPT, b) Average Monthly flow, c) 60 years average annual flow variations and d) Average Monthly temperature of Kunhar watershed over three meteorological stations.

The Kunhar River Basin extends from 34.2° to 35.1°N and 73.3° - 74.1°E, covering a catchment area of 2650 km^2^. River Kunhar originates from Babosar lake, and it flows to Mangla reservoir after merging in Neelam River in Muzaffarabad (Fig. 1a). The Kunhar River's major tributary is 166 miles in length, 642 m above sea level is the watershed's lowest elevation point, while 5106 m above sea level represents its highest elevation point. The Kunhar River meanders through Bata Kondi, Jalkand, Kaghan, Naran, Balakot, Kawai, and Garhi Habibullah before eventually joining the Jhelum River near Rara. Abundant in algal flora, the waters of the Kunhar River support a diverse array of aquatic life. Agricultural Land-Generic, Forest-Deciduous, Pasture, Barren, and Snow are some of the vegetation types found in the Kunhar basin [32]. Seventy five percent of the basin is covered by a predominant soil type called leptosol medium (swat database code 3712), ten percent of the basin consists of Combisole fine (swat code 3673), Combisol medium (swat code 3672) and five percent of the basin is covered by Glacier (swat code 6998). Fig. 2 (d) depicts the geographic distribution of soil types.

The Kunhar watershed has a 53 % average slope. Slopes between 0 and 10 % cover only 3 % of the basin's surface, while slopes between 30 and 60 % cover about 42 %. As measured by the Ghari Habibullah stream gauge, the annual mean flow of the Kunhar River was 102.6 m^3^/s. Three meteorological stations, at Narran, Balakot, and Muzaffarabad were used to gather the climate data (Table 1). The LULC (till Oct 2020) of the KRB consists of a wide range of ecosystems. Agricultural, wooded, grazing, desolate, and snowy are just a few examples (Fig. 1c).

**Table 1:** KBR hydrological, meteorological data.

Station	Lat. (◦)	Long. (◦)	Altitude (m)	Record Period	Data Source
Hydrological stations					
**Shoran, Muzzaffarabad**	34.38	73.47	704	1971–2020	PMD/K-Water
Meteorological stations					
**Balakot**	34.55	73.35	995	1971–2020	PMD
**Muzzaffarabad**	34.37	73.47	704	1971–2020	PMD
**Naran**	34.89	73.66	2422	1971–2020	PMD

### 2.2 Data acquisition

To execute the SWAT model, essential prerequisites include Digital Elevation Model (DEM), soil map, Land Use/Land Cover (LULC) map, and climate data. Using Arc SWAT in the ArcMAP10.3.1 environment, the SRTM DEM (Shuttle Radar Topography Mission Digital Elevation Model) (Fig. 1b) having spatial resolution of 30 × 30 m was utilized to delineate the watershed of the KRB (Arc SWAT and its extensions can be freely acquired from the website of the United States Department of Agriculture and Agricultural Research Service (USDA-ARS) (https://swat.tamu.edu/software/arcswat/). LULC data was collected from the ESRI (Environmental Systems Research Institute) LULC database having spatial resolution of 10 × 10 m. Annual data for the period 1990–2020 is included in the ESRI LULC dataset, which is comprised of 10 LULC classes. The FAO World Soil Digital Map was used to compile soil data.

The daily climate data was provided by Pakistan Meteorological Department (PMD) and Water and Power Development Authority (WAPDA). Data collected on daily basis from 1961 to 2020 from PMD and WAPDA encompasses the highest (Tmax) and lowest temperatures (Tmin), precipitation at two weather stations (Naran and Balakot), and flow discharge at Patrind dam recorded at the Shoran station. Fig. 2 shows the historical data from 1960 to 2020.

### 2.3 Acquisition of climatic data

Nine general circulation models were utilized to acquire projected climate data. Analysis of projections from the Earth System Grid Federation (ESGF), the World Climate Research Program (CMIP), and the Climate Change Knowledge Portal (CCK) was conducted to assess the anticipated changes in precipitation, maximum temperatures, and minimum temperatures over the coming decades. After data was extracted, it was subsequently bias adjusted for the future. The CMhyd software employed the linear scaling (L.S) method for bias correction. Extracted data from the climate model was linearly related to observed climate data in LS. Bias correction of ppt: formulae are given in Equations (1), (2)). Similarly, Equations (3), (4)) represent mathematical models for temperature bias correction. The approach has been used successfully in other climate studies (Akbar and Ghewala 2020 [32], Lafoon et al., 2013 [33])

• **Precipitation**

(1) $$P_{h}^{+}=P_{h}\left(d\right)\;\frac{am\left(P_{ob}\left(\mathrm{d}\right)\right)}{am\left(P_{h}\left(d\right)\right)}$$

(2) $$P_{f}^{+}=P_{f}\left(d\right)\;\frac{am\left(P_{ob}\left(\mathrm{d}\right)\right)}{am\left(P_{h}\left(d\right)\right)}$$

• **Temperature**

(3) $$T_{h}^{+}=T_{h}\left(d\right)+\text{am}\;\left(T_{\text{ob}}\left(d\right)\right)\;–\;\text{am}\;\left(T_{h}\left(d\right)\right)$$

(4) $$T_{f}^{+}=T_{f}\left(d\right)+\text{am}\;\left(T_{\text{ob}}\left(d\right)\right)\;–\;\text{am}\;\left(T_{h}\left(d\right)\right)$$

Where P shows precipitation, T represents temperature, ob is used for observed, h = historical run, f = future run, a = average, d = day, m = monthly, ^**+**^ shows the bias-corrected data.

### 2.4 The SWAT model

Hydrological models assess how climate change affects water resources at various scales encompassing global, regional, watershed, and ecosystem levels (Van Grieensven et al., 2012 [34], et Bresiannial.2015 [35]). River flow and runoff can be modeled across various scales, ranging from local basins to a global level (Sitterrson et al., 2017 [36,37]). SWAT (Arnold et al., 1998 [38,39], the Hydrologic Simulation Program Fortran (HSFP:US EPA 2019 [40] and the Hydrologic Engineering and Modelling Centre Hydrologic Modelling system HEC-HMS2000 [41]) are all useful models for assessing and predicting hydrological changes [42]. A physical-based continuous-time, based on processes river basin and semi-distributed model, SWAT's original objective was to forecast the enduring effects of climate change and land-use management procedures on water, agricultural chemical outputs, and sediments in composite river basins of moderate to large size ([38,39]). Based on soil type, slope classes, and land use, SWAT splits watersheds into smaller simulated regions known as hydrological response units (HRUs). The model predicts hydrology for each Hydrological Response Unit (HRU) by solving the water balance equation, which includes daily factors such as percolation, precipitation, evapotranspiration, runoff, and return flow. To forecast surface runoff, the model utilizes two approaches: (a) the Green and Ampt method and (b) the Natural Resources Conservation Service Curve Number (CN) method. Storage routing methods in conjunction with a crack-flow model are used to forecast the percolation across each soil layer. In SWAT, three techniques are used to assess evapotranspiration: (i) Penman Monteith, (ii) Priestley-Taylor and (iii) Hargreaves. The Muskingum method or the adjustable storage coefficient technique is employed to compute the flow routing in the river channels ([38,39]).

The model can simulate variations in discharge in response to changes in both the immediate surface environment and the underlying meteorological or hydrologic variables. The water balance equation [43] is the conceptual backbone of the model's implementation.

(5) $$SW_{t}=SW_{0}+\sum_{i=1}^{i}\left(R_{day}-Q_{surf}-E_{a}-W_{see_{P}}-Q_{gw}\right)\;$$

Where SW_0_ is primary soil moisture content, SW_t_ = final soil moisture content while R_day_ = precipitation at day i, Q_surf_ = surface runoff at day i, Ea = evapotranspiration at day i, W_seep_ = soil flow depth on day i, Q_gw_ = depth of groundwater runoff on day i, and t = time. Water and time measurements are expressed in millimeters and days, respectively.

### 2.5 Configuration of the SWAT model

**Fig. 3:**
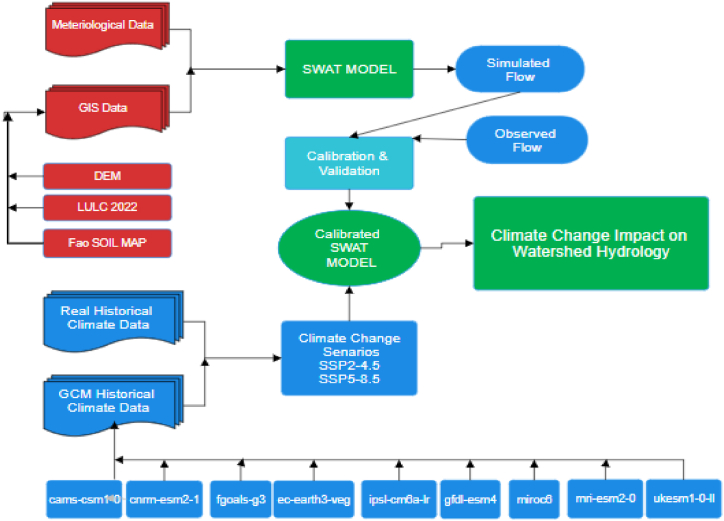
Flowchart of the approach underlying this investigation.

The built up of the SWAT model involved the following steps: (i) Developing DEM, delineating watersheds and sub-watersheds; (ii) Analyzing Hydrological Response Units (HRUs) and elevation bands; (iii) Generating a soil map; (iv) Executing the model and configuring the parameters; and (v) Conducting calibration, validation, and uncertainty analysis. (Fig. 3). The model version of SWAT 2013 was used for this research because it provides increasingly precise simulation results when new modules are added, calculation methods are revised, and the range of applicability is broadened. The model was using the same methodology that proved successful in earlier research (Ahmed et al., 2012 [44]; Rehman et al., 2013 [45]; Haguma et al., 2014 [46]; P.J.Y and R.A.Km 2014 [47]; Koycigiz and Buyokyildiz 2019 [48]; Shange et al., 2019 [49]; Haidar et al., 2020 [50]; Musiee et al., 2020 [22]). The SWAT model simulates two steps in the hydrological process: runoff generation and overland flow concentration. The KRB was split into 277 HRUs and 23 sub-basins for current research.

Calibration of the SWAT model was conducted over a 21-year period (1990–2010), followed by validation for the subsequent 10 years (2011–2020). In addition, the years 1985–1989 served as a model warm-up. SWAT-CUP was utilized for sensitivity analysis. The recent past was selected as the calibration and validation period to reflect current changes in the basin. Because the basin has undergone such rapid transformation in recent decades, we avoid using historical data from before the year 1990. There is no computational procedure that can replace in-depth physical knowledge of the watershed for calibrating a physically based model like SWAT, and all inputs need to be maintained within an acceptable range of uncertainty (Arnold et al., 2012 [38]). Calibration and validation of a model over a longer time frame is preferable.

### 2.6 General circulation models and climate change scenarios

The GCM is a simple and effective way to evaluate the global warming process. Modelling accuracy for the same basin or region varies depending on the CMIP6 model's process, initial condition setting, resolution, etc. Table 2 outlines the model's name and fundamental characteristics.

**Table 2:** Specifications of the 9 GCMs utilized in CMIP6.

S.No	GCM	Country	Institution	Resolution
1	cams-csm1-0	China	Chinese Academy of Meteorological Sciences, Beijing	1.1° x 1.1°
2	cnrm-esm2-1	France	Centre National de Recherches Meteorologiques (CNRM)	0.5° x 0.5°
3	ec-earth3-veg	Sweden	EC-Earth consortium, Rossby Centre, Swedish Meteorological and Hydrological Institute/SMHI, Norrkoping, Sweden	0.7° x 0.7°
4	fgoals-g3	China	Chinese Academy of Sciences, Beijing, China	1.3o x 1.0o
5	gfdl-esm4	USA	National Oceanic and Atmospheric Administration, Geophysical Fluid Dynamics Laboratory, Princeton, NJ, USA	1.3° x 1.0°
6	ipsl-cm6a-lr	France	Institute Pierre Simon Laplace (IPSL), Paris, France	2.5o x 1.3o
7	miroc6	Japan	Japan Agency for Marine-Earth Science and Technology (JAMSTEC), Kanagawa, Japan	1.4° x 1.4°
8	mri-esm2-0	Japan	Meteorological Research Institute (MRI), Tsukuba, Ibaraki Japan	1.1° x 1.1°
9	ukesm1-0-ll	UK	Met. Office Hadley Centre, Exeter, UK	1.9° x 1.3°

Shared Socioeconomic Pathways (SSPs) are used instead of CMIP5's representative concentration pathways (RCPs). The Shared Socioeconomic Pathways (SSPs) examine the relationship between radiative forcing and societal development to formulate the next set of climate change scenarios. Even though the new scenario anticipated by CMIP6 has a similar radiative forcing to that predicted by CMIP5, the main distinction between SSPs and RCPs is carbon dioxide and other gaseous emission pathways, and their combined paths. The SSP2-4.5 scenario represents a middle position, balancing between the extremes of societal vulnerability and radiation coercion. Consequently, it is categorized as an intermediate scenario. The SSP5-8.5 scenario is the only option to reach the 8.5 (W/m^2^) level of man-made radiative forcing by 2100.

## 3 Results and discussion

### 3.1 Sensitivity analysis of variable parameters

The applicability of hydrological models and parameter sensitivities can be explored using the sequential uncertainty fitting-2(SUFI-2) algorithm within SWAT-CUP. SWAT-CUP is equipped with various tools, including Parameter Solution (ParaSol), Generalized Likelihood Uncertainty Estimation (GLUE), and Sequential Uncertainty Fitting-2 (SUFI2), to assess parameter sensitivity and conduct uncertainty analysis. Among these algorithms, SUFI-2 has gained significant popularity and is widely utilized for parameterization, calibration, validation, sensitivity, and uncertainty analysis (Abbaspour et al., 200 [51]; Sao et al., 2020) [52]. Based on the literature [50,53,54] SWAT-CUP identified 13 of the most delicate parameters, which are given in Table 3. The SUFI-2 technique was employed for iterative simulations to optimize model parameters, ensuring the attainment of optimal results. For this purpose, we employ the t-statistic and associated p-value generated by the SUFI-2 algorithm. A greater value of t-Stat suggests that the parameters are more sensitive. The sensitivity importance is indicated by the p-value. When the p-value gets closer to 0, the sensitivity becomes more significant. Parameters of the models were defined in terms of their physical meanings and the initial ranges were provided based on previous research (Table 3) [53,55].

**Table 3:** Discharge's range of sensitive parameters for model Calibration.

Ranking	Parameters	Description	t-Stat	p-Value	Adjusted Value	Max. And min Value
1	v_ESCO.hru	Evaporation Soil Compensation Factor	−15.89	0	0.892	0.8–1
2	v_CH_N2.rte	The Manning n coefficient for the primary communication channel	−4.125	0	0.094	0–0.3
3	v_SLSUBBSN.hru	Average slope length	−4.052	0	48.15	10–150
4	v_GW_DELAY.gw	Groundwater delay	−1.632	0.070	105.55	0–500
5	v_ALPHA_BF.gw	Alpha factor of base flow	−1.562	0.073	0.35	0–1
6	v_SMFMX.bsn	Melt factor for snow	−1.585	0.078	2.55	0–20
7	v_CH_K2.rte	Hydraulic conductivity of the primary channel	−1.523	0.115	16.88	5–130
8	v_GWQMN.gw	Minimum depth of water in a shallow aquifer needed for the return flow	−1.466	0.118	1300	0–5000
9	v_SMTMP.bsn	Temp of snow's melting base	−1.432	0.125	8.85	−20 - 20
10	r_SOL_K (1).sol	Soil saturated hydraulic	−1.365	0.146	0.60	−0.8–0.8
11	r_SOL_AWC (1).sol	Capacity of soil's top layer to hold water	−0.885	0.322	−0.089	−0.2–0.4
12	v_GW_REVAP.gw	Groundwater re evaporation coefficient	−0.756	0.539	0.093	0–0.2
13	r_CN2.mgt	Curve number	−0.652	0.601	−0.167	−0.2–0.2

According to Table 3, the most delicate variable is the Evaporation Soil Compensation Factor (ESCO). The evaporation of soil water is dominated by ESCO. When ESCO rises, less soil water is lost to evaporation. As a result, there will be more runoff. The average slope length (SLSUBBSN) and the Manning's n coefficient for the main channel (CH_N2) are the second and third most sensitive model parameters, respectively. The impact of terrain and geomorphology on precipitation is largely reflected in SLSUBBSN. Changes in ESCO, SLSUBBSN, and CH_N2 severely affect runoff yield in the KRB using the T-Stat value, but the remaining 10 parameters have a considerably smaller impact.

### 3.2 SWAT model applicability analysis

#### 3.2.1 Evaluation indicators assessing model efficiency

Nash-Sutcliffe efficiency (NSE), percent bias (PBIAS), and the R^2^ statistic were used to evaluate the hydrological models' accuracy. The R^2^, coefficient of determination, shows how collinear the simulated and measured data are. The NSE shows how well the predicted and actual curves match. R^2^ and NSE values, ranging from 0 to 1, indicate less inaccuracy with higher values. The PBIAS shows the typical percentage difference between actual and simulated flows and the typical slope of the simulated flows. The acceptable range of PBIAS values is −15 %–15 %, with a positive number indicating an underestimation and a negative value indicating an overestimation. (Akbar and Gheewala, 2020 [56]).

To evaluate how effectively a model simulates observed trends in an important output response over time, the NSE can be computed using Equation (6) [57].

(6) $$NSE=\left[1-\frac{\sum_{i=0}^{n}{\left(Q_{o.i}-Q_{s,i}\right)}^{2}}{\sum_{i=1}^{n}{\left(Q_{o,i}-Q_{o,m}\right)}^{2}}\right]$$

(7) $$\text{Pbias}=\left[\frac{\sum_{\text{i}=0}^{\text{n}}\left({\text{Q}}_{\text{o},\text{i}}-{\text{Q}}_{\text{s},\text{i}}\right)}{\sum_{\text{i}=0}^{\text{n}}{\text{Q}}_{\text{o},\text{i}}}\right]*100$$

(8) $$R^{2}=\left[\frac{\sum_{i=1}^{n}\left(Q_{o,i}-Q_{o,m}\right)\left(Q_{s,i}-Q_{s,m}\right)}{\sqrt{\sum_{i=1}^{n}{\left(Q_{o,i}-Q_{o,m}\right)}^{2\;}}\;\sqrt{\sum_{i=1}^{n}{\left(Q_{s,i}-Q_{s,m}\right)}^{2}}}\right]$$

Equations (6)–(8) use the number of simulated and observed pairings (n) as a parameter. The observed flow is denoted by *Q*o, the simulated flow by *Q*s, the average observed flow by *Q*o,m, and the simulated average flow by *Q*s,m.

**Fig. 4:**
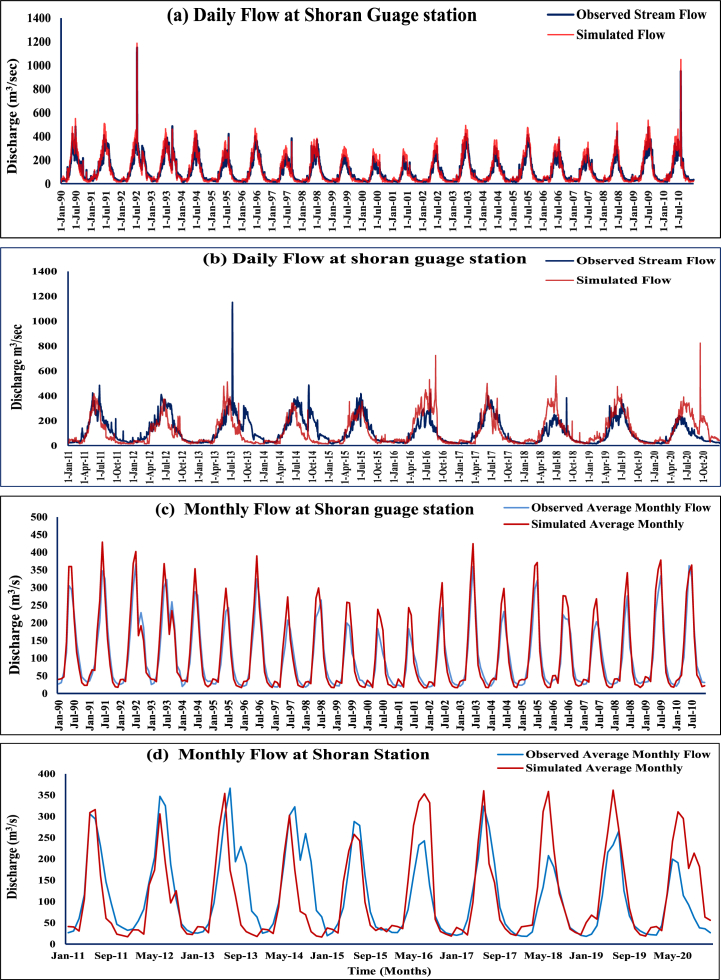
Comparison of observed and simulated flow rates during (a) Calibration, (b) Validation on Daily bases and (c) Calibration, (d) Validation on Monthly basis.

If NSE is more than 0.50 and PBIAS is maintained at 25 % for streamflow, objective function performance on monthly time base calibration is outstanding (Moriasi et al., 2007 [58]). Good simulation is defined as an NSE of 0.75 or higher, whereas acceptable simulation is defined as an NSE of 0.36 or higher (Nash & Sutcliffe, 1970) (Kachroo 1992 [59]). Daily time series plots and monthly time series plots illustrate that the observed and projected discharges correspond well during the calibration (1990–2010 and validation (2011–2020) phases (Fig. 4).

**Fig. 5:**
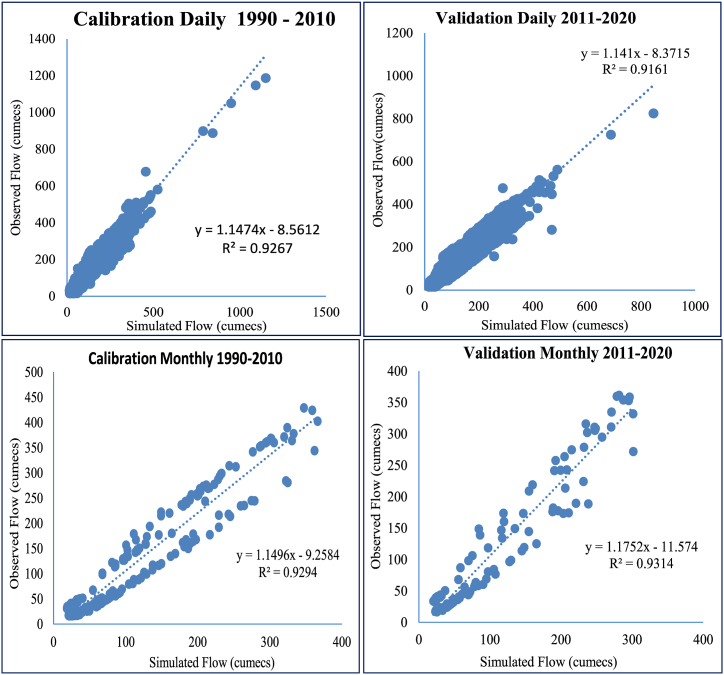
Fitting relationship between the daily and monthly simulated and measured runoff.

Fig. 5 shows R^2^ values for both the calibration (Daily) and validation (Monthly) phases, for all nine models, the data points are concentrated around the 1:1 line (observed discharge vs simulated discharge). High, medium, and low flows can be compared using flow duration curves, which can be derived from either observed or simulated data using hydrologic models [60].

**Fig. 6:**
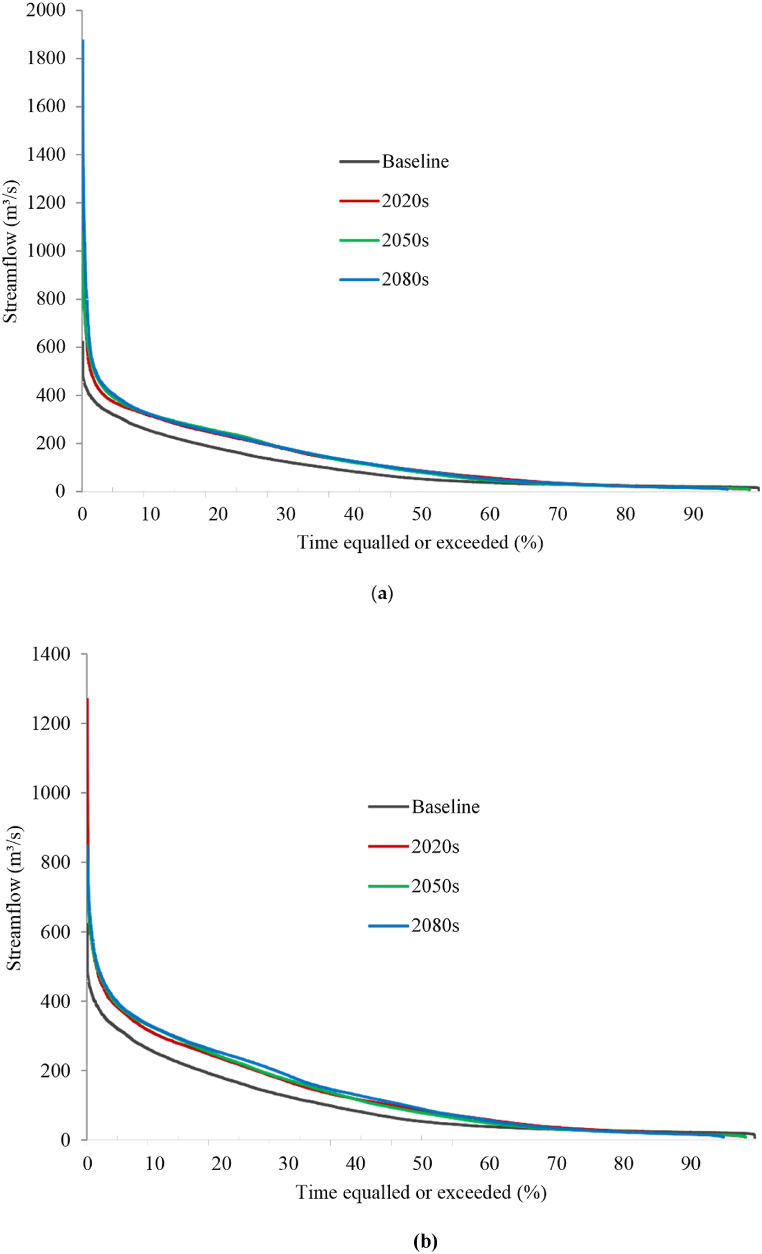
Flow duration curves under (**a**) SSP 2–4.5 and (**b**) SSP 5–8.5 scenarios in the Kunhar River basin.

Fig. 6 illustrates the comparison of flow duration curves for the Shoran hydrometric station, displaying the baseline period (1990–2020) and three future periods (2020s, 2050s, and 2080s) under SSP2-4.5 and SSP5-8.5 scenarios. This comparison indicates a potential increase in the probability and magnitudes of flow in the future within the Kunhar basin under both scenarios.

Table 5 outlines the projected changes in high flow (Q5), median flow (Q50), and low flow (Q95) for the 2020s, 2050s, and 2080s concerning the baseline under both scenarios. In the Kunhar basin, Q5 and Q50 are anticipated to rise in all three future periods under both scenarios, with increments ranging from 17 % to 52 % (Q5) and 43 %–84 % (Q50). This elevation in mean flow is likely attributed to the projected increase in mean annual precipitation, and the rise in high flow is probably linked to augmented summer precipitation during the monsoon season, consistent with the findings of Mahmood and Babel.

The Flow Duration Curve (FDC) is a cumulative frequency curve illustrating the percentage of occurrences, where a specific discharge or flow rate was equaled or exceeded throughout a given time period, according to Equation (9),

(9) P(%)=M/(n+1)×100

P stands for the possibility that a specific flow will be met or exceeded, presented as a percentage. M denotes the assigned position in the ranking, while n signifies the number of events during the record period, with both being dimensionless).

Table 4 summarizes the key performance metrics obtained from SWAT model simulation results for daily and monthly discharge where for daily discharge simulation, the NSE values are greater than 0.75 for calibration and validation periods, while the respective R^2^ values are 0.88 and 0.90. For monthly discharge simulation, the SWAT model has significantly higher NSE, R^2^, and KGE values compared to daily discharge simulation.

**Table 4:** The SWAT model's calibration and validation performance indicators.

Temporal Scale	Period	NSE	R^2^	PBIAS
Daily	Calibration	0.87	0.92	−6.53
	Validation	0.86	0.91	−6.33
Monthly	Calibration	0.87	0.92	−6.08
	Validation	0.91	0.93	−6.73

**Table 5:** Percent future changes in low, median, and high flows with respect to the baseline (1990–2020) under SSP 2–4.5 and (**b**) SSP 5–8.5 scenarios in the Kunhar River basin.

SSP2-4.5 Scenario	1990–2020 (m³/s)		2020s		2050s		2080s
	G. Habib		G. Habib		G. Habib		G. Habib
*Q*_5_	321		19		23		29
^*Q*^50	60		71		56		81
^*Q*^95	22		−23		−24		−19
**SSP5-8.5 Scenario**							
*Q*_5_		321		20	22		25
^*Q*^50		55		66		52	91
^*Q*^95		22		−22		−24	−22

*Q*_5_, high flow; *Q*_50_, median flow; *Q*_95_, low flow.

### 3.3 Analysis of the average monthly streamflow at shoran station, muzaffarabad over a period of five years

**Fig. 7:**
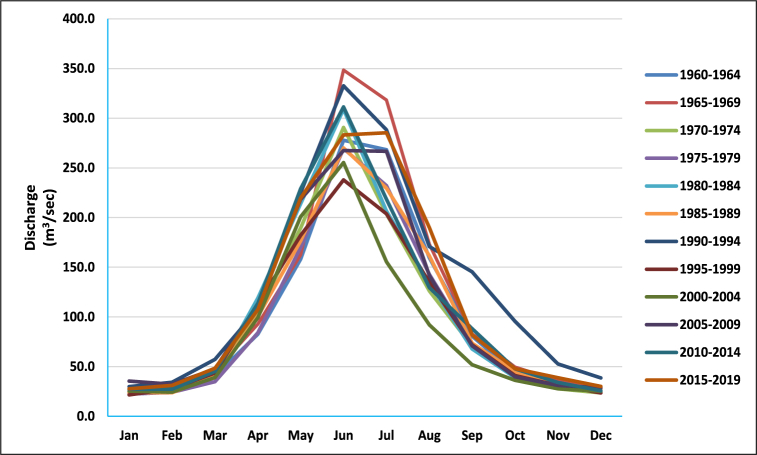
Mean monthly flow at Shoran station, Patrind, for a 50-year period (1960–2019).

The streamflow baseline data (1960–2019) is equally divided in spans of five years (Fig. 7). For each time period, the average monthly runoff is calculated. Following an increase in May, June experienced a noted decline in the average monthly flow. The lowest peak flow occurred during 1995–1999 and the highest value of the peak flow was recorded in 1965–1969 followed by the 1992 and 2010 devastating floods.

## 4 Future temperature and rainfall projections

**Fig. 8:**
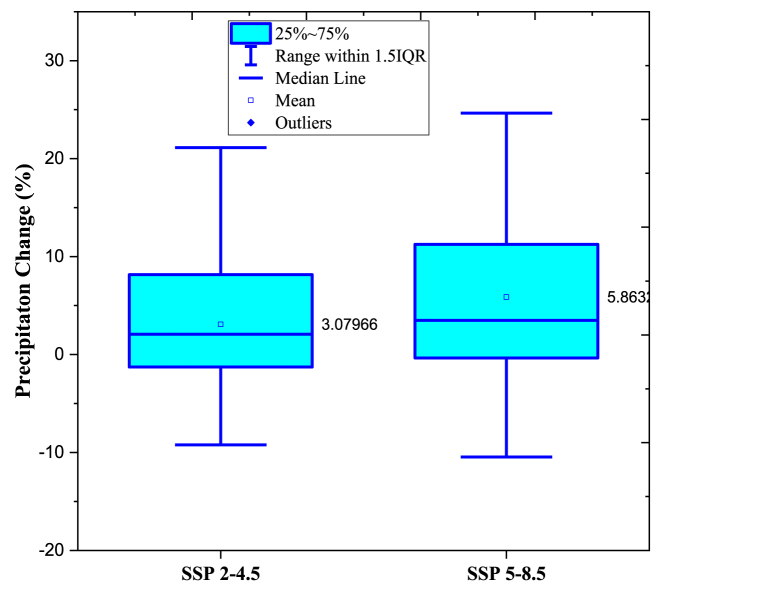
The boxplot of the % variations in precipitation for SSP2-4.5 and SSP5-8.5 in nine different GCMs vs baseline period.

Rainfall variability directly influences the hydrological cycle and the runoff process, rendering it a valuable indicator of climate change [61]. The baseline period is from 1960 to 2020, while the prediction period is 2021–2099. The mean annual rainfall for the reference period was 1597.20 mm. Percentage changes in annual average precipitation for 2020–2099 period, Fig. 8 illustrates the values in comparison to the baseline for both SSP2-4.5 and SSP5-8.5. More than half of the GCMs forecast an increase in precipitation under SSP2-4.5 emission scenario, varying from a low of −9.23 % to a high of 21.12 % for the future (2021–2099). For the SSP5-8.5 emission scenario, the range of projected values for precipitation increases across all GCMs is (−0.354 %, 11.254 %), with the lowest projected value being −10.45 % and the highest increase being 24.63 %. The mean annual precipitation in the KRB varies little from one SSP scenario to the next, but it does exhibit an upward tendency (Fig. 8), while other emission scenarios have precipitation increase rates that are slower than historical rates, with the SSP2-4.5 emission scenario having the lowest rate of increase while for SSP5-8.5 emission scenario, there is a larger rise in precipitation.

**Fig. 9:**
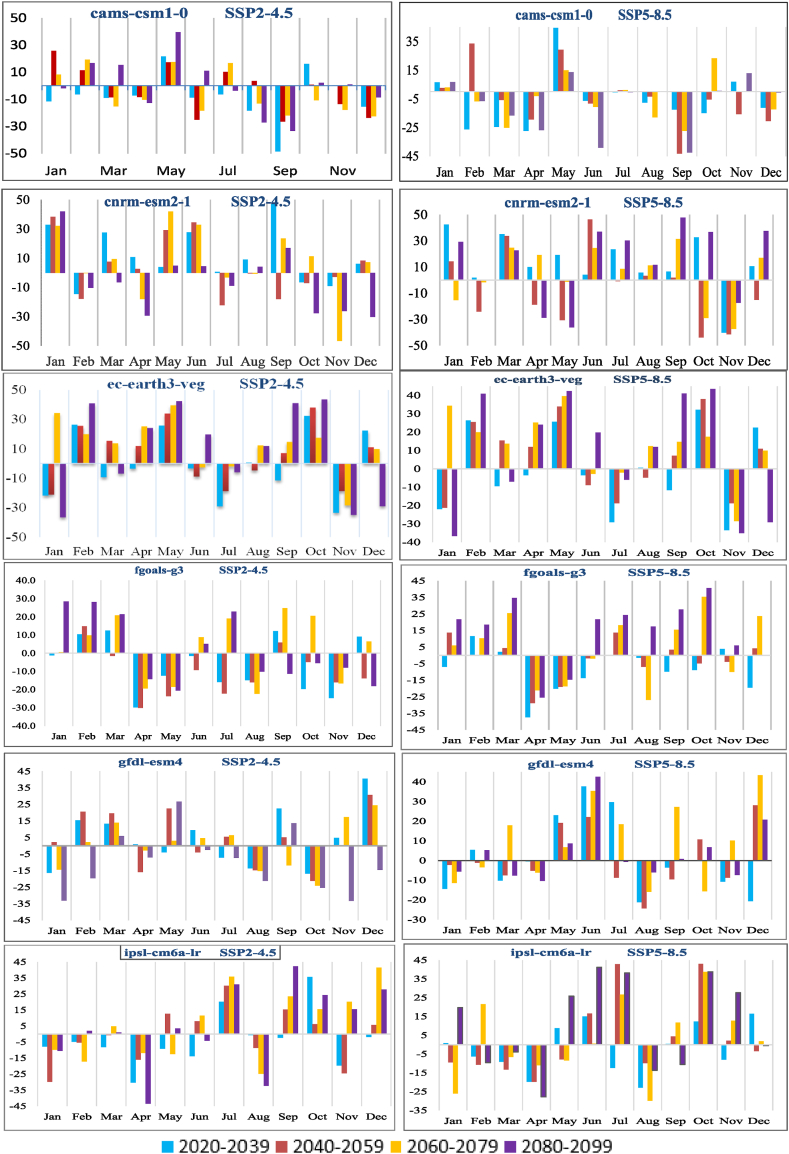

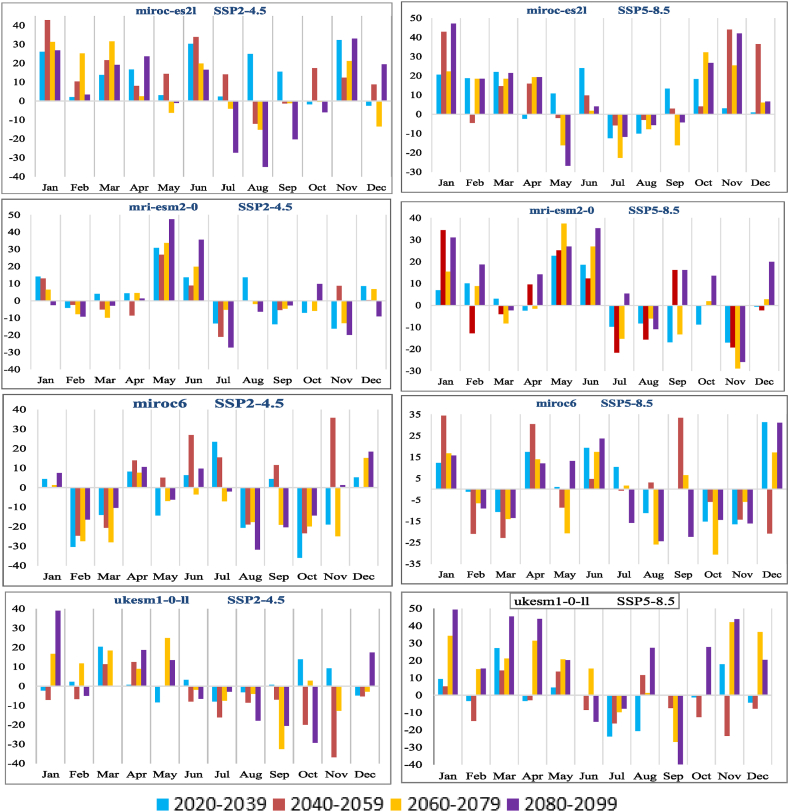
The projected variation (% change, y-axis) of the annual precipitation in the future the time 2021–2099.

Monthly precipitation increases for the two SSPs scenarios were simulated in nine GCMs to evaluate near, mid and far projections. In Fig. 9, the percentage changes in precipitation for the near, mid, and far future across all nine General Circulation Models (GCMs) are shown. When using CAMS-CSM1-0, precipitation increases significantly in warm weather and in the late autumn, with increases of up to 28 % in the mid future and 40 % for the early monsoon season in the far-future and a 50 % decrease in the early autumn season in the SSP 2–4.5 emission scenario. Precipitation from the early monsoon is predicted to rise by 45 % in the near future under the SSP 5–8.5 emission scenario, even though CAMS-CSM1-0 predicts a 39 % fall in the hot period over the far future and a 45 % rise in the early prediction period. CNRM-ESM2-1 has a forecast of 42.41 % rise in the mid-winter for the near future and 47.9 % rise in fall in the far future period while more than 40 % decrease for most of the future time periods under SSP5-8.5 emission scenario. EC-EARTH3-VEG estimates an incredible 65.96 % shift in winter for mid future and an increase of 47 % in the start and end of monsoon the mid and late future under SSP5-8.5 emission scenario. GFDL-ESM4 model predicted an increase of 30–40 % in the summer and winter under SSP5-8.5 while up to 30 % decrease in winter, summer and fall under SSP2-4.5. IPSL-CM6A-LR, MRI-ESM2-0, MIROC6 and UKESML-0-LL predict decreasing trend under SSP2-4.5 scenario. There is a 35 % increase in the warm weather predicted by MRI-ESM2-0, MIROC6 and UKESML-0-LL under SSP5-8.5 in the mid and far periods. Considering watershed records, we can anticipate a rise in both the volume and intensity of liquid precipitation during the rainy season and snowfall during the cold season [62].

**Fig. 10:**
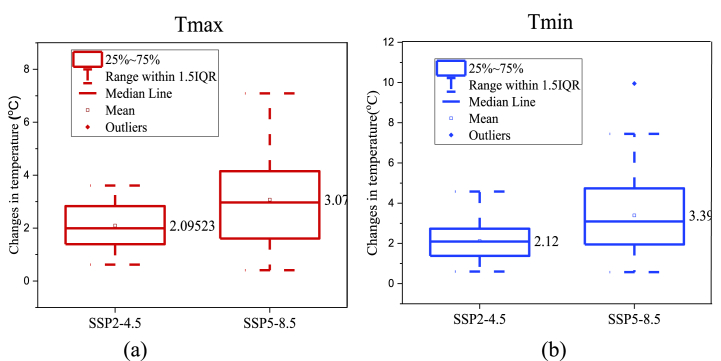
Box plot presentation of temperature variations of 9 GCMs in (a) Maximum temperature and (b) Minimum temperature under SSP2-4.5, and SSP5-8.5.

**Fig. 11:**
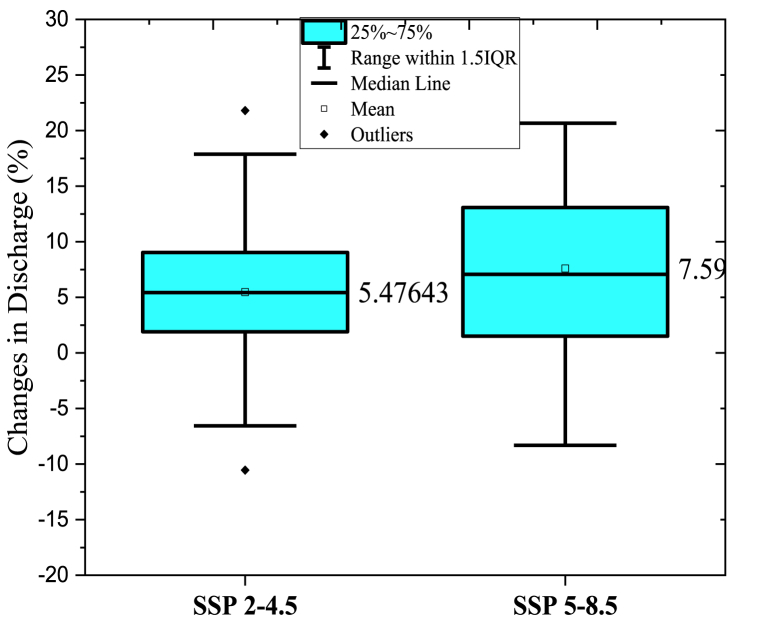
The percentage change in discharge compared to the baseline is examined across nine Global Climate Models (GCMs) under the SSP2-4.5 and SSP5-8.5 scenarios.

Temperature indirectly influences runoff through its impact on surface evapotranspiration [29]. According to the IPCC, there is a (1–3%) rise in global mean evaporation for every 1 °C increase in temperature [29]. For the baseline, yearly maximum temperatures average 20.63 °C and lows average 12.19 °C. The baseline period from 1990 to 2020 is being compared under the two SSPs emission scenarios to assess the variations in annual mean high and low air temperatures (Fig. 10). The mean temperature of the KRB is predicted to rise significantly in future. All nine GCMs show that the lowest and extreme air temperatures will rise over the next few decades (Fig. 11). Under the SSP5-8.5 emission scenario, the maximum air temperature will increase by 3.07 °C, with a range of 1.60 °C–4.15 °C, while under the SSP2-4.5 emission scenario, the maximum air temperature will increase by an average of 2.09 °C, with a range of 1.39 °C–2.83 °C. Under SSP 2–4.5, there is an average increase of 1.38 °C (1.12 °C–2.73 °C) in the minimum air temperature, whereas under SSP 5–8.5, there is an average reduction of 3.39 °C (1.94 °C–4.74 °C) in the lowest temperature. Based on historic precipitation and temperature, the KRB will continue to have hot and humid hydrothermal conditions.

### 4.1 Climate change impacts on runoff

Annual projected flow percentage of KRB changes (Fig. 11) by all nine GCMs reflected that the discharge will increase by an average value of 5.42 % and (25–75) % of values of all GCMs will range in (1.90–9.04) % under SSP2-4.5 emission scenario and by an average value 7.60 % and (25–75) % of all GCMs will range in (1.51–13.08)% in the SSP5-8.5 emissions scenario. The average monthly and seasonal variations in runoff from KRB from 2021 to 2099 are shown in Fig. 14. Peak monthly discharge values range from 368.74 m^3^/s in 2099 to 271.22 m^3^/s in 2059, with the maximum runoff found in 2099 under SSP2-4.5 emission scenario. The average monthly peak runoff increases to 387.54 m^3^/s in (2020–2039) with the SSP5-8.5 emission scenario.

Fig. 14 illustrates the comparison between the mean monthly discharge during the baseline period (1990–2020) and the mean monthly discharge in future periods (2020s, 2050s, 2080s and 2099) to analyze temporal shifts and the magnitude of peak flows at Shoran Station. For this location, distinct delays and rises in peak flows were projected in all four future periods under both scenarios. The forecasted shift in peak flows was from June to July in some GCMs, accompanied by a 10%–25 % increase under both scenarios. This indicates that the basin is not only expected to experience a heightened frequency and magnitude of floods, as mentioned in earlier sections, but also a shift in the occurrence of these floods from June to July. The ec-earth3-veg model is more likely to predict an increase in winter and spring flows relative to baseline than a substantial drop in summer flows relative to baseline. In the SSP5-8.5 emission scenario, the maximum flow variability was determined (Fig. 14). In the SSP2-4.5 and SSP5-8.5 emission scenarios, the early summer flow is predicted to raise by up to 28.6 % in all future periods, but in the fgoals-g3 scenario, it is predicted to decline by up to 7.47 %. According to mri-esm2-0, the future monsoon flow might increase by 59.28 % under SSP2-4.5 and by just 29.43 % under SSP5-8.5 emission scenarios. Future increases of up to 41.70 % were predicted by CAMS-CSM1-0 for the SSP2-4.5 emission scenario and increases of up to 43.92 % were predicted for the SSP5-8.5 scenario. In addition, ec-earth3-veg projects a rise of up to 48.53 % during mid-monsoon periods in the middle-future, whereas the SSP5-8.5 emission scenario forecasts a decline to 17.3 %. It was observed that in CAMS-CSM1-0, ec-earth3-veg and mri-esm2-0 the peak flow is shifting to early summer while in fgoals-g3, gfdl-esm4, ipsl-cm6a-lr the peak flow appeared to be shifted from June to July and august. Analyses of water flow have shown that both summer and winter water flows have increased significantly. This could stem from substantial shifts in monsoon months and winter evapotranspiration within General Circulation Models, prompted by the significant rise in spring temperatures.

Earlier snowmelt, influenced by rising temperatures and changes in precipitation patterns and intensity, could result in an earlier peak flow rate. Additionally, the possibility of increased rainfall may contribute to elevated water flow levels in rivers and streams. Some of the general circulation models predicted that high flows could shift to the spring and summer's peak flow could shift to July in both SSP scenarios. The average annual flow, however, has the potential to rise in the far and near future, but not in the near future. SSP5-8.5 allows for a little greater streamflow than SSP2-4.5. Some general circulation models also predict that by the mid-future, streamflow will be lower than the baseline.

Future runoff and precipitation trends in the basin are expected to follow the same general pattern across time. Runoff increases in each case with higher levels of precipitation, demonstrating a strong positive link between the two. This demonstrates a positive link between changes in precipitation and basin runoff, while changes in temperature have only an indirect effect on runoff [63]. In the future, the KRB could experience flood levels that are substantially higher than those of the past. This phenomenon is not helpful to manage the future water resources in the KRB since it will increase the pressure to control flooding during the wet season. In the future, water resources management presents new issues for local planning authorities. The SWAT model was executed using GCMs, although it is probable that the runoff process in the watershed is far more spatially variable than the atmospheric process. The downscaling, emission scenarios, climate model, and hydrological model used to estimate runoff all have their own set of unknowns. Runoff generation and water supplies are also significantly impacted by human activities such the growth of urban impermeable surfaces, reservoir operations, and other changes in land use need further research [64,65].

**Fig. 12:**
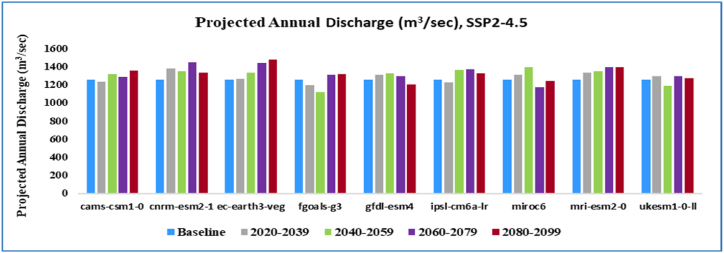
Average yearly flow at Shoran station (2020–2099) under SSP2-4.5 of Nine GCMs.

**Fig. 13:**
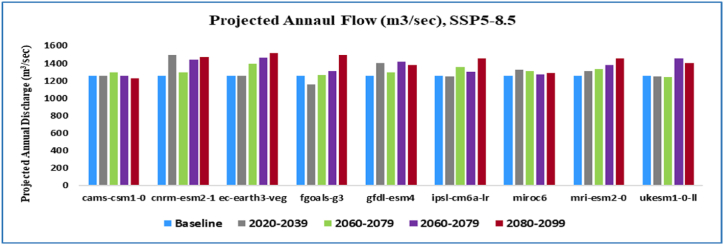
Average yearly flow at Shoran station (2020–2099) under SSP5-8.5 of Nine GCMs.

The KRB's average annual streamflow variations by climate change are given in Fig. 12, Fig. 13. The SSP2-4.5 scenario predicts a 10 % increase in annual runoff, while the SSP5-8.5 scenario predicts a 19.50 % increase. In the SSP2-4.5 scenarios, annual runoff decreases by 11 % by mid-century, while in the SSP5-8.5 scenarios, it rises by 11.5 %. In the far future (2071–2099), the SSP2-4.5 and SSP5-8.5 scenarios forecast an 18 % and 21 % increase in annual runoff, respectively as compared to the baseline period.

**Fig. 14:**
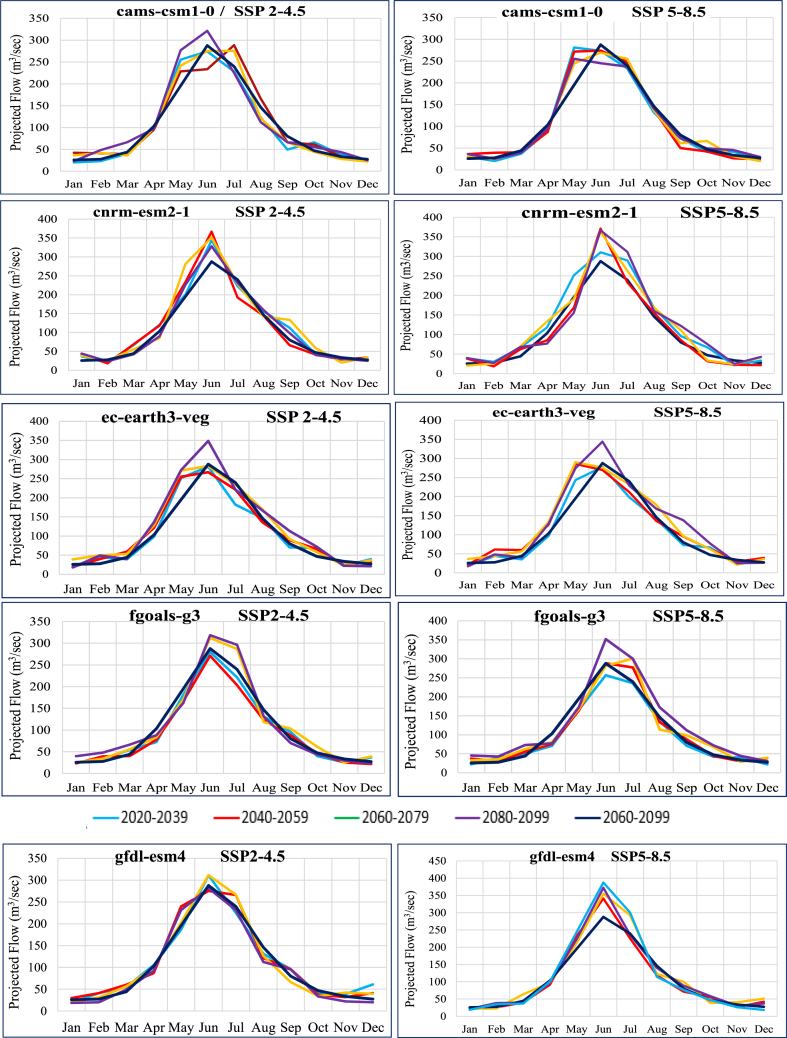

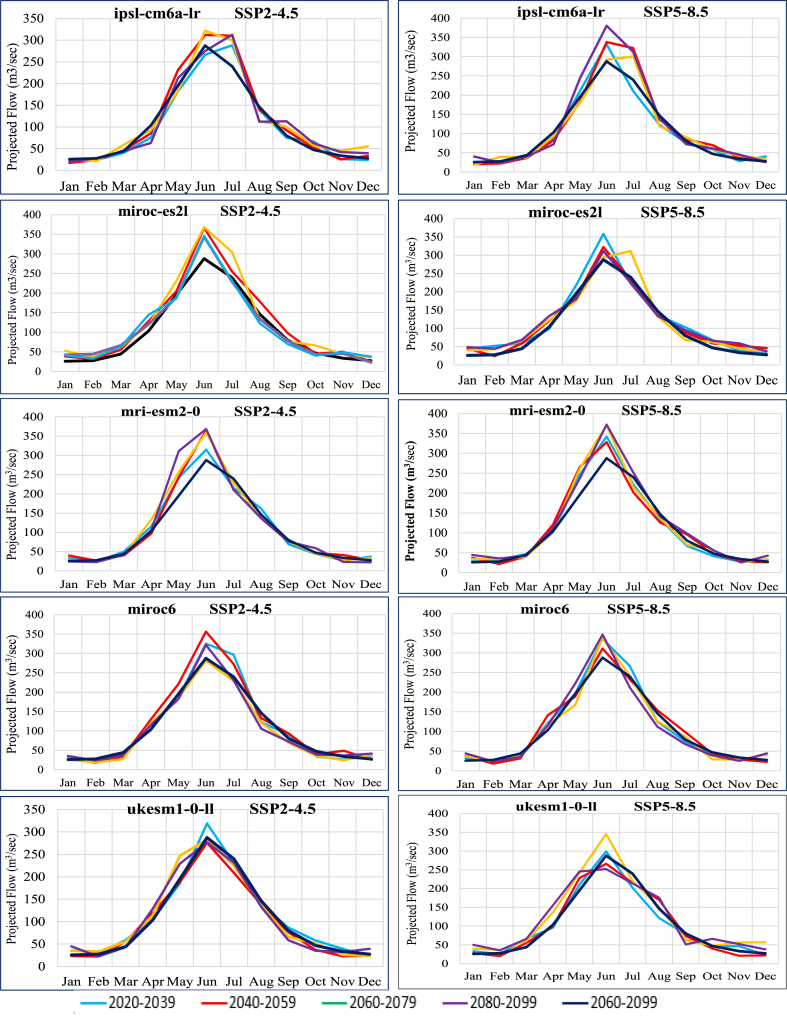
Projected flows (m^3^/month) by nine GCMs under CMIP6 emission scenarios

## 5 Discussion

The flow predictions for the climate conditions are found to be sensitive to climate forecast and modelling parameters, suggesting that comparing our results to those of another research in the same basin is useful. As a result, the previous studies proved to be the most useful for our research. Each research used unique climate data and hydrological models to provide vastly different forecasts for the future. River flows are found to be highest in the spring and lowest in the winter, as determined by Baber et al. (2016) [12]. However, Mahmod et al. (2016) [13] observed an inverse trend in their study of annual mean flow during different seasons. Ghulam Nabi et al. (2022) [64] predict annual average flow at Ghari Habibullah will increase between 2021 and 2040, decrease between 2041 and 2070, and then increase again between 2071 and 2099. Our results are more in line with those of Ghulam Nabi et al. (2022) [64] but we utilized CMIP6 models boasting improved physical, chemical, and biological process representation and higher spatial resolution compared to CMIP5 models (Stouffer et al., 2017; Zhu & Yang 2020) [66]. The predicted and baseline flow regimes are compared in Table 6, which displays yearly and seasonal differences. Future changes in the flow regime are difficult to predict because different research reflects different baseline periods. Baseline flow (26 m^3^/s) predicted to increase by 44.12 %, 69.11 %, and 110.9 % in the near, middle, and far futures for the RCP 8.5 scenario by Babar et al. (2016) [12], whereas Mahmod et al. (2016) [13] reported a 2 %, 11 %, and 2 % change in baseline flow (25 m^3^/s) in the near, middle, and far futures, respectively. Table 6 shows that projections for future flows indicate a rise of 112 % in the short-term, 151.2 % in the intermediate-term, and 193.8 % in the long-term according to research by Ghulam Nabi et al. (2022) [67], while according to research by Mehmood et all.2016 shows decrease in most of the future projections under both scenarios. Hydrological model projections depend heavily on the details of the climatic data and calibration parameters with which they are made. Our research found the highest percent change in winter flows are 76.56, 77.24, 61.28, 29.50 in near, mid, far-mid and far future periods respectively by mri-esm2-0 under SSP2-4.5 emission scenario, while SSP5-8.5 emission scenario reflects 10.22, 3.56, 24.67 and 51.44 percent changes for respective future winter flows.

**Table 6:** Results comparison with earlier research conducted on the same watershed.

KRB projection flows (cumecs) at Ghari HabibUllah in the future (% change from the baseline) by HEC-HMS model (Mehmood et al., 2016) [13].					(Nabi et al., 2022) Projection flows (cumecs) for the future of the KRB at Ghari HabibUllah by the SWAT model and MRI-CGCM3 GCMs (percent variation from the baseline) [57].				Future projection flows (cumecs)of KRB at Shoran station, Muzaffarabad by SWAT and MRI-ESM2-0 climate models (% variation from the baseline)				
									Present Study				
	1961–1990	2020	2050	2080	1979–2010	2020	2050	2080	1961–2020	2020–2039	2040–2059	2060–2079	2080–2099
**A2 Scenario**					**Scenario RCP 4.5**								
Winter	25	0	−6	8	26	112.2	97.6	66	26.9	76.56	77.23	61.3	29.5
Spring	110	−14	−26	−12	111	10.1	4.2	37.9	114.23	18.66	10.88	25.62	33.58
Summer	226	38	46	49	215	6.3	−33.1	−2.9	224.6	2.83	6.05	8.24	6.4
Autumn	58	109	107	126	53	60.9	−21.9	17.9	53.53	−12.88	3.01	−6.95	−2.04
Annual	105	33	30	43	102	21	−13.2	15	104.82	5.75	7.5	5.31	0.78
**B2 Scenario**					**Scenario RCP 8.5**								
Winter	25	−2	−11	2	26	112.8	151.2	193.8	26.9	10.22	3.57	−24.67	51.44
Spring	110	−16	−28	−10	111	3.9	27.3	60.4	114.23	14.54	24.32	17.16	13.77
Summer	226	38	93	46	215	−13.8	−24.7	−29.7	224.6	3.45	−2.8	7.22	13.4
Autumn	58	97	91	131	53	10.3	37	9.7	53.53	−15.96	6.06	−6.18	11.4
Annual	105	29	24	42	102	2.1	8.7	14.2	104.82	1.3	3.74	7.46	18.6

Changes in LULC are another indirect way in which climate change impacts regional water resources. The LULC map (Fig. 1c) indicates that the main causes of land-use change in the KRB are urbanization, shifts in agricultural development, and other human activities. Recent LULC changes are only minor causes to climate change effects. The government of Pakistan intends to prioritize rural agriculture as a growth sector in the future, and it actively supports initiatives to protect farmland. The existing research suggests that the overall area of cropland may not be drastically affected by future climate change. Since the effect of land use shifts due to climate change is uncertain, we predict the catchment's future water resources by focusing only on precipitation and temperature changes.

SP2-4.5 and SSP5-8.5, the former shows a greater temperature increase, while the latter shows a greater amount of precipitation and discharge. In Fig. 10, (25–75) % of the GCMs used in this study, the maximum temperature in the SSP5-8.5 emission scenario varies from 1.6 °C to 4.15 °C while in SSP2-4.5 emission scenario it varies from 1.39 °C to 2.83 °C. The minimum temperature raise is also more in SSP5-8.5 emission scenarios. Precipitation and runoff are directly proportional, with more rain leading to more runoff in each situation. There is a positive correlation observed between changes in precipitation and basin runoff. [29], but changes in temperature have only an indirect effect. To plan for future run-of-river hydroelectric projects, it is likely that the Kunhar River Basin's discharge will continue to rise. Managing water resources in the future of the KRB will be negatively impacted by the increase in the pressure to control floods during the wet season [68]. In every emission scenario, it was found that all GCM results consistently align in the direction of flow changes. Throughout the projected periods, there were slight variations observed in the river streams. The water flow data indicate a significant increase in water flows during the hot season, accompanied by an overall reduction in water flows during the cold season. This could be attributed to the significant rise in spring temperatures, influencing the monsoon months and causing substantial shifts in winter evapotranspiration according to GCMs.

Several factors, such as downscaling, the climate model, emission scenarios, and the hydrological model, contribute to the uncertainty in runoff estimates. Runoff production and water availability are also significantly impacted by changes in urban impervious surfaces, reservoir operations, and other LULC variations [69]. These aspects, however, are outside the scope of the present investigation and require more investigation.

## 6 Conclusions

The SWAT model accurately predicted the runoff on a monthly and daily basis. NSE and R^2^ values for the discharge simulation are both more than 0.75 in both the calibration and validation phases. Consistent hydrographs were produced by the gfdl-esm4, miroc6, and ukesm1-0-ll, making them more suitable for use in model calibration and validation. Under the various SSPs scenarios, the KRB should expect a rise in both precipitation and temperature. Under the SSP2-4.5 emission scenario, annual precipitation is expected to rise by 3.07 % (−9.22 %, 21.12 %) compared to the baseline (1990–2020) (Fig. 8), while under the SSP5-8.5 emission scenario, annual precipitation is expected to rise by 6.68 % (1.61 %, 12.75 %). Maximum air temperatures are expected to rise by 2.09 °C (0.62 °C–3.61 °C) under scenario SSP2-4.5 and by 3.07 °C (0.41 °C–7.09 °C) under scenario SSP5-8.5, while minimum temperatures are predicted to rise by 2.09 °C (0.6 °C–4.58 °C) under scenario SSP2-4.5 and by 3.39 °C (1.94 °C–7.45 °C) under scenario SSP5-8.5.

Projected future climate change indicates an increasing trend in estimated runoff within the Kunhar River basin. The multi-year average runoff in the basin is expected to increase by 5.42 % (−6.55 to 17.07 %) and 7.59 % (−8.3 to 20.67 %) under the SSP2-4.5 and SSP5-8.5 scenarios, respectively, for the period from 2020 to 2099 compared to the baseline. Until the end of this century, we may see a decline of 13 % in summer runoff relative to the baseline, while increases of 77.24 % (in scenario SSP2-4.5 in the mid future) and 40.09 % (in scenario SSP5-8.5 in the far mid future) in winter and spring streamflow are possible. In the far future (in ec-earth3-veg under SSP5-8.5) and the near future (in cnrm-esm2-l under SSP5-8.5), the average annual discharge could increase by 20 %, while in the mid-future (in fgoals under the scenario SSP2-4.5), it is more likely to drop by 10 %.

This research has the potential to serve as a starting point for exploring the influence of climate change on hydropower. This is attributed to the notable fluctuations in projected streamflow, which can consequently affect the hydropower potential within the Kunhar River basin. Moreover, the study offers valuable insights, particularly regarding the near future, that can aid policymakers in the water sector when making decisions related to Sustainable Development Goals, specifically Clean Water & Sanitation (SDG 6), Climate Action (SDG 13) and SDG15 (Life on Land) [70]. Moreover, the establishment of a new run-of-the-river hydropower plants in the region has the potential to create employment opportunities for the local community [[71], [72], [73]]. This initiative can contribute to achieving the indicator SDG 1.1, which aims to eradicate extreme poverty for all people everywhere, falling under the broader goal of SDG 1 (No Poverty).

## Achievements and further recommendations

It is important to introduce the results with a note of caution, highlighting the areas requiring additional research and incorporating gaps identified in previous studies. These achievements are:

The first constraint is associated with the underlying assumptions of the climate model and emission scenario. The SSP scenarios, in which CMIP6 accounts for mitigation and adaptation to a variety of societal challenges and enables researchers to assess the impact of these challenges, have been incorporated into the debate over the reliability under dynamics of the socioeconomic trend utilized for projecting these emissions of a changing world in 2018. Also, SSPs make assumptions about the world's population, educational opportunities, economic growth, urbanization, developments in technology, and other demand-driving factors like changes in lifestyle, produced better results than RCPs in the previous related research in the same area.

Secondly, the KRB features a wide range of hydrogeological and topographical conditions. It was not possible to capture the complete range of hydro climatic details in the KRB with only two hydro climatic stations. Data limitation can affect the hydrological model performance. In this study we included a new hydrological gauge at Shoran station in Patrind dam which was operational in 2017 and produced close results to simulated flow as compared to the previous studies. Also, 300 automated weather stations are planned for Pakistan with cooperation of the World Bank-funded project will be completed in almost three years which will enhance reliable weather data in future (PMD, 10.13039/501100010055Hydro Met and Climate Services Project, November 2022).

Thirdly, we used nine RCMs and bias-correction strategy to lower the uncertainty in our climate projection, as was recommended by previous research. Future investigations on the relationship between hydropower and global warming could benefit from the findings of this research. The hydropower potential within the KRB could face potential impacts due to significant variations in projected streamflow. To maximize energy output, hydropower operators may need to modify their reservoir management strategies by considering the changing patterns of inflow. The gap between water availability and demand and water supply for runoff river dams will be alleviated by an increase in runoff. However, variations in extremes due to excessive rainfall could contribute to the flooding threat. Climate change-related flooding problems will likely provide new difficulties for flood control. A further recommendation in this research is to investigate sediment yield projections under CMIP6 scenarios and machine learning applications.

## Limitations of the study

In this current research, the assessment of climate change effects on the water resources within the Kunhar River basin involved the utilization of nine GCMs to address uncertainties associated with GCM outputs. This study assessed the performance of 35 GCMs from CMIP6 in replicating APHRODITE rainfall patterns in Mainland South-East Asia (MSEA) spanning from 1975 to 2014. The ranking of GCMs was determined using Compromised Programming (CP) and three spatial statistical metrics. Additionally, Jenk's natural break classification was utilized to identify the most suitable subset of GCMs for MSEA. The basin has only one meteorological station available, indicating a scarcity of data for the region. This data scarcity may result in reduced performance levels of a hydrological model during the calibration and validation processes. Throughout the simulation period, land cover and soil properties were assumed to remain constant, and such an assumption can impact the streamflow projections in the basin. **Author Contributions:** The research was carried out by A.W., H.J., F.J. and K.M. A.W. was involved in data collection, data analysis, the creation of the initial draft, and the implementation of the SWAT model. H.J. and F.J. contributed to the conceptualization of the study. K.M. was involved in supervision, reviewing and editing the manuscript, and acquiring funding.

## Funding

Not applicable.

## Data availability statement

The datasets used and/or analyzed during the current study available from the corresponding author on reasonable request.

## Credit author statement

Muhammad Hidayat Jamal: Writing – original draft, Supervision, Software, Resources, Funding acquisition, Conceptualization. ABDUL WAHEED: Writing – review & editing, Writing – original draft, Visualization, Validation, Software, Resources, Methodology, Investigation, Formal analysis, Data curation, Conceptualization. Muhammad Faisal Javed: Writing – review & editing, Visualization, Supervision, Resources, Conceptualization. Khairul Idlan Muhammad: Provided CMIP6 Data of the GCMs and Bias Correction.

## Declaration of competing interest

The authors declare that they have no known competing financial interests or personal relationships that could have appeared to influence the work reported in this paper.
